# Heartland Virus Transmission, Suffolk County, New York, USA

**DOI:** 10.3201/eid2712.211426

**Published:** 2021-12

**Authors:** Alan P. Dupuis, Melissa A. Prusinski, Collin O'Connor, Joseph G. Maffei, Kiet A. Ngo, Cheri A. Koetzner, Michael P. Santoriello, Christopher L. Romano, Guang Xu, Fumiko Ribbe, Scott R. Campbell, Stephen M. Rich, P. Bryon Backenson, Laura D. Kramer, Alexander T. Ciota

**Affiliations:** New York State Department of Health, Slingerlands, New York, USA (A.P. Dupuis II, J.G. Maffei, K.A. Ngo, C.A. Koetzner, L.D. Kramer, A.T. Ciota);; New York State Department of Health, Albany, New York, USA (M.A. Prusinski, C. O’Connor, P.B. Backenson);; Suffolk County Department of Health Services, Yaphank, New York, USA (M.P. Santoriello, C.L. Romano, S.R. Campbell);; University of Massachusetts, Amherst, Massachusetts, USA (G. Xu, F. Ribbe, S.M. Rich);; State University of New York at Albany School of Public Health, Albany (L.D. Kramer, A.T. Ciota)

**Keywords:** Heartland virus, HRTV, arboviruses, viruses, ticks, white–tailed deer, Amblyomma americanum, lone star tick, surveillance, transmission, vector–borne infections, zoonoses, Suffolk County, New York, United States

## Abstract

During 2018, Heartland virus RNA was detected in an *Amblyomma americanum* tick removed from a resident of Suffolk County, New York, USA. The person showed seroconversion. Tick surveillance and white–tailed deer (*Odocoileus virginianus*) serosurveys showed widespread distribution in Suffolk County, emphasizing a need for disease surveillance anywhere *A. americanum* ticks are established or emerging.

Heartland virus (HRTV; *Phenuviridae*, *Bandavirus*) is an emerging human pathogen initially isolated from patients in Missouri, USA, during 2009 (*1*). Since then, >50 known human cases have been identified in Arkansas, Georgia, Illinois, Indiana, Iowa, Kansas, Kentucky, Missouri, North Carolina, Oklahoma, and Tennessee (*2**–**5*). *Amblyomma americanum*, the lone star tick, has been implicated in HRTV transmission and maintenance (*6**–**8*). Small–sized and medium–sized mammals and ground dwelling birds, such as wild turkeys (*Meleagris gallopavo*), serve as hosts for immature ticks. Adult ticks feed primarily on large mammals, such as coyotes (*Canis latrans*) and white–tailed deer (*Odocoileus virginianus*). Ticks at all 3 active developmental stages will bite humans (*9*). Serologic evidence in mammal hosts, including white–tailed deer, indicates that HRTV is distributed primarily in the Midwest and southeast United States, as well as the northeastern Atlantic coast (*10**–**12*).

During August 2018, New York State Department of Health (NYSDOH) epidemiologists were notified that HRTV RNA was detected in an *A. americanum* nymph removed from a resident of Long Island, New York, USA. This infected tick was tested at the University of Massachusetts (https://www.tickreport.com).

In response, the NYSDOH and Suffolk County Department of Health Services conducted tick surveillance and performed HRTV serologic analysis on the person from whom the tick was removed. Analysis was also performed for a hunter–harvested white–tailed deer in Suffolk County.

## The Study

Officials with the NYSDOH and Suffolk County Department of Health Services contacted a Long Island, New York, resident for a follow–up investigation after receiving notification that a tick removed from the resident and submitted for comprehensive pathogen testing was positive for HRTV RNA. The resident, a man in his 60s, removed the tick on August 8, 2018, and recalled having a low–grade fever (maximum temperature 100.5°F) and fatigue for 5 days beginning on August 15, 2018. He noted no other symptoms.

Serum was provided at multiple time points for serologic analysis. We tested serum samples by using a standard 90% plaque reduction neutralization test (PRNT_90_) for HRTV (strain M12–66) (*8*), provided by the Centers for Disease Control and Prevention. We tested samples at Wadsworth Center, NYSDOH, and results were confirmed by the Centers for Disease Control and Prevention. Neutralizing antibody titers were 1:20, 1:160, and 1:160 for samples collected at 8, 50, and 96 days after symptom onset (15, 57, and 103 days after removal of the tick), respectively, indicative of a recent infection with HRTV.

We initiated standardized drag and flag sampling of host–seeking *A. americanum* ticks on public lands for arbovirus surveillance during 2016, before HRTV detection. We found that 132 pools (containing 475 nymphs and 437 adults) from 4 Suffolk County locations were negative for HRTV by real–time reverse transcription PCR using established protocols (*8*). During 2018, tick surveillance at 5 locations yielded 102 pools (969 adults); all were negative for HRTV.

Increased efforts during the public health investigation conducted on August 23 and 24, 2018, yielded an additional 113 *A. americanum* ticks (92 larvae and 21 nymphs) from a location where tick exposure potentially occurred. All ticks collected during the investigation were negative for HRTV. No ticks were found during sampling of the property surrounding the residence of the case–patient.

**Figure 1:**
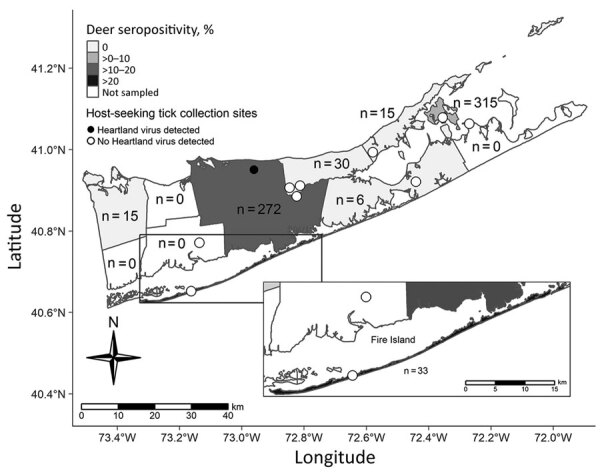
Tick collection sites in study of heartland virus transmission, Suffolk County, New York, USA. Numbers within townships indicate sample size of deer tested for neutralizing antibody.

During 2019 and 2020, tick surveillance in the towns of Brookhaven and Riverhead yielded 1,123 pools of *A. americanum* ticks (2,788 adults and 6,728 nymphs) (Figure 1). We found that 3 pools of unengorged nymphs collected from the Brookhaven site on June 14 (n = 1) and June 24 (n = 2), 2019, and 2 pools of unengorged nymphs collected from the same location on July 25 and August 5, 2020, were positive for HRTV RNA. We isolated virus from 2 tick pools after incubation on Vero cells. We found that testing of >1,100 *Ixodes scapularis* ticks (199 pools) collected during the surveillance campaign in Suffolk County, during 2018–2020, were negative for HRTV.

**Figure 2:**
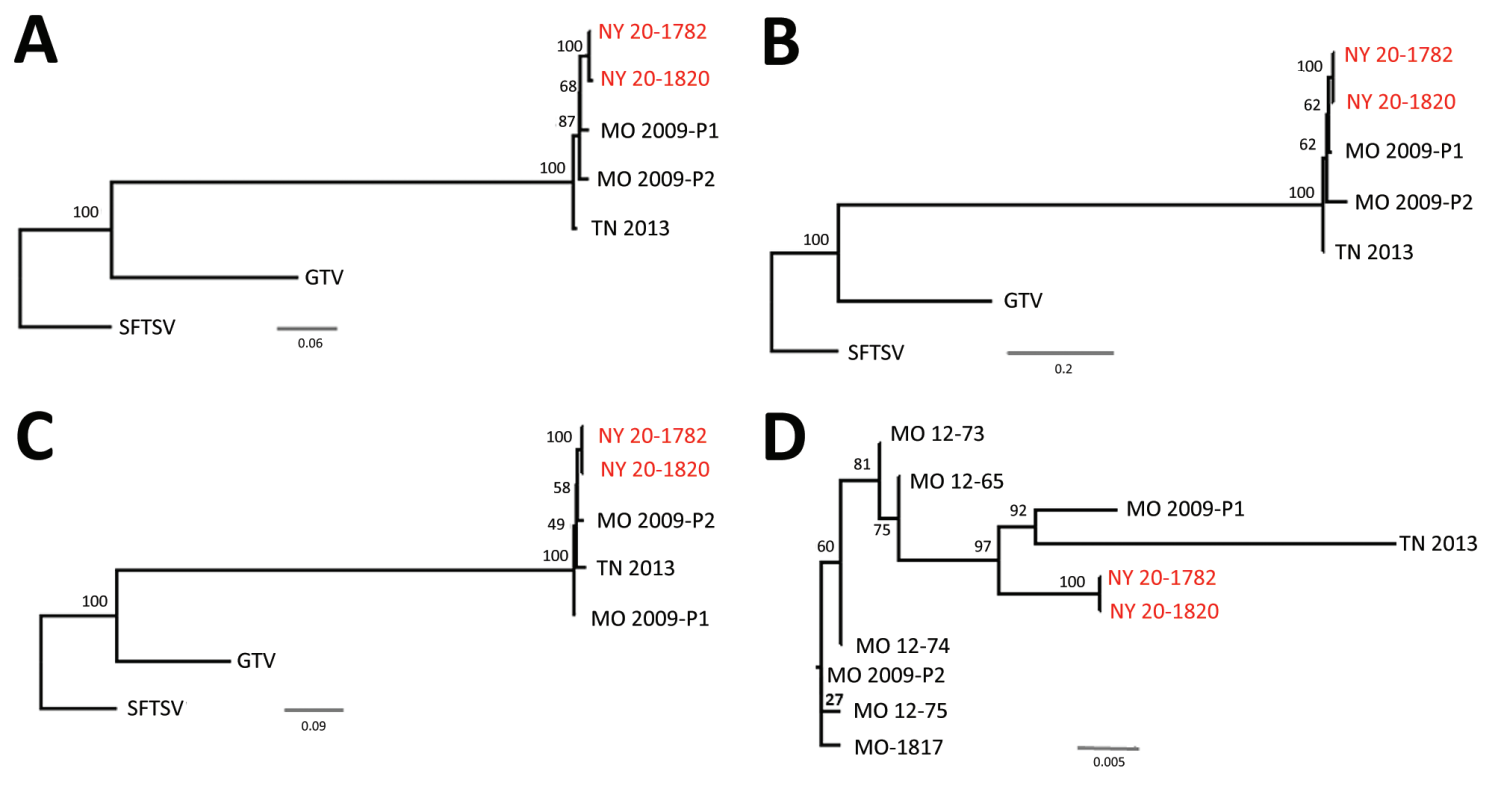
Phylogenetic relationship among Heartland virus isolates, Suffolk County, Long Island, New York, USA. Separate alignments of large segments (A), medium segments (B), small segments (C), and partial nonstructural sequences (D) were created with MAFFT in Geneious version 11.1.5 (https://www.geneious.com). Maximum–likelihood analyses were completed with RAxML (https://cme.h–its.org) using 1,000 bootstraps. Bootstrap values are indicated at each node. Phylogenetic trees for each segment were rooted to SFTSV strain HB154 (GenBank accession nos. JQ733560–62). Guerta virus strain DXM was included as an additional outgroup (GenBank accession nos. 328591–93). New York isolates from this study (red text), together with the 3 previously available full–genome sequences (MO 2009–P1 [patient 1, GenBank accession nos. JX005842, 4, 6]; MO 2009–P2 [patient 2, GenBank accession nos. X005843, 5, and 7]; and TN 2013 [TN, GenBank accession nos. J740146–8]), were included in these analyses (panels A, B, and C). Six additional partial sequences available for a 606–nt region of the nonstructural protein gene (GenBank accession nos. C466555, KC466560, KC466561, KC466562, KC466563, and MT052710) are indicated in an unrooted maximum–likelihood tree in panel D. Scale bars indicate nucleotide substitutions per site. GTV; Guerta virus; SFTSV, severe fever with thrombocytopenia syndrome virus.

We extracted RNA from isolates by using established protocols (*13*). We developed primer pairs to amplify the small, medium, and large RNA segments by using a One–Step Superscript III Reverse Transcription PCR with Platinum Taq (Life Technologies, https://www.thermofisher.com) (Table 1). We performed 3 separate reactions using 5 μL of RNA, 1 μL of polymerase, and 0.2 μmol/L final concentration of primer pairs in a total reaction volume of 50 μL. We amplified products with the following thermocycler conditions: 55°C for 30 min; 94°C for 2 min; 40 cycles at 94°C for 30 s, 57°C for 45 s, and 68°C for 4 min; and a final extension at 68°C for 10 min. Amplicons were visualized by electrophoresis on a 1% agarose gel. Products were pooled and purified for next–generation sequencing at the Wadsworth Center, NYSDOH, Applied Genomics Core. We prepared libraries by using the Nextera XT Kit (Illumina, https://www.illumina.com) and performed sequencing using the MiSeq Illumina platform; we analyzed sequences by using Geneious Prime Software (https://www.geneious.com) (Table 2; Figure 2).

We conducted serologic testing of hunter–harvested white–tailed deer blood submitted for arbovirus serosurveys by using PRNT_90_, as described (*14*). We screened 686 serum samples at a dilution of 1:20 for neutralizing antibodies to HRTV (Figure 1) and serially diluted positive serum samples for endpoint titers. Overall, 9.8% of the deer were seropositive and had titers ranging from 1:20 to >1:640; 76% of the seropositive deer had titers >1:20. We tested 1,641 *A. americanum* ticks collected from 145 sampled deer for HRTV RNA but did not detect any virus.

## Conclusions

Evidence of widespread HRTV transmission was demonstrated throughout Suffolk County, New York. Consistent with previous studies, *A. americanum* ticks were implicated in local transmission of HRTV. All positive pools were nymphal stage ticks, including the tick originally submitted for testing at the University of Massachusetts. Tick minimal infection rates ranged from 0% to 1.1%. It is unclear whether flat nymphs had acquired the virus as larvae feeding on viremic hosts, through cofeeding transmission, or transovarially because each of these modes has been demonstrated in the laboratory (*7*).

The lack of HRTV detection in adult ticks is notable if one considers that collections occurred at the same site across 3 seasons. Higher numbers of positive nymph pools were observed in Missouri, where 53/60 HRTV–positive tick pools collected at sites near the first described human cases were nymphs (*6*). Complete genome sequence analysis of the HRTV strains isolated during this study showed >98% amino acid and >93% nucleotide identities to the original strains isolated from patients in Missouri during 2009 (*1*) and a strain isolated in Tennessee during 2013 (*2*).

White–tailed deer are a sensitive sentinel model for many arboviruses, given their abundance, limited home range, and the frequency on which they are fed upon by hematophagous arthropods (*10**,**11**,**14*). Approximately 10% of the deer sampled during this study were seropositive against HRTV. Our serologic testing strategy differed from those of previous studies by using a more stringent PRNT_90_. Suffolk County deer seropositive rates were similar to those reported in Vermont (10%), Maine (11%), and Florida (4%) deer (*12*). The rates are lower than those reported for deer tested in midwestern and southeastern states, areas with burgeoning populations of *A. americanum* ticks (*10**,**11*). To date, no competent vertebrate host, including deer, has been implicated in HRTV amplification (*15*).

Results from this study emphasize the need to include HRTV in surveillance programs wherever *A. americanum* ticks are distributed. Furthermore, clinicians should be aware of this pathogen and the potential for overlapping symptomologies (fever, fatigue, and loss of appetite) with other tickborne infections. Providers should request HRTV testing for patients who have clinical symptoms, including leukopenia and thrombocytopenia, and a history of tick exposure or travel to regions where *A. americanum* ticks are reported.
